# Patient-Centered Measure Development and Spanish Validation Exemplar

**DOI:** 10.3928/24748307-20190925-01

**Published:** 2019-11-05

**Authors:** Robin T. Higashi, Shannon B. Juengst

## Abstract

Traumatic brain injury (TBI) often results in cognitive impairments that require investigators to consider language accessibility of survey instruments, clinical evaluations, and other research tools. We describe an iterative language validation process for the Behavioral Assessment Screening Tool (BAST) and BAST Spanish version (BAST-ESP), consisting of two phases: (1) achieving an accessible literacy level for English-speaking people with TBI and (2) translating, validating, and cognitively testing the BAST-ESP for Spanish-speaking people with TBI. Investigators recruited scientific experts and members of the target populations to adapt and test the surveys. Modifications to original survey instruments included simplified semantic structures, enhanced conceptual clarity, rephrased idiomatic expressions, and rewording to bridge cultural differences in linguistic connotation. Findings from participants in focus groups and cognitive interviews confirmed accuracy and ease of comprehension and informed further adjustments and content relevant to the specific target populations. We demonstrate the importance of a systematic adaptation and validation process to develop a lower-literacy instrument appropriate for people with cognitive deficits and to enhance the BAST-ESP beyond translation alone. This article, along with a previously published article about BAST content validity process, provides a road map for other investigators to conduct systematic adaptation of scientific instruments for low-literacy and non–English-speaking populations. **[*HLRP: Health Literacy Research and Practice*. 2019;3(4):e243–e249.]**

People with traumatic brain injury (TBI) experience unique cognitive deficits requiring investigators to consider language accessibility when developing and using self-reported measures, clinical evaluations, and other research tools. Yet, clinicians and researchers often fail to properly assess and adapt health information and study materials, despite it being recommended patient-centered care and ethical practice according to the National Institutes of Health (National Cancer Institute, 1995), Institute of Medicine (Kindig, Panzer, & Nielsen-Bohlman, 2004), and American Medical Association (Weiss, 2007)

There is a well-known link between low literacy and adverse health outcomes, including timely receipt of care, communication with providers, and medication adherence (Berkman et al., 2011; Keller, Wright, & Pace, 2008; Zhang, Terry, & McHorney, 2014). Low health literacy also affects the quality of health research if study materials are not adapted to accommodate people with limited capacity for reading comprehension and communication of ideas. This may result in erroneous conclusions about statistical differences, which may have been attributable to a lack of comprehension of the study materials rather than a true clinical difference.

The development process for the Behavioral Assessment Screening Tool (BAST) specifically addresses this gap in language accessibility for both populations with low literacy and cognitive impairment. Investigators developed the BAST for long-term monitoring of behavioral and emotional symptoms after TBI (Juengst, Terhorst, Dicianno, Niemeier, & Wagner, 2019). To be effective as a community-based symptom-monitoring tool, people must read and respond to survey items independently, magnifying the importance of language accessibility. The BAST was meticulously tested to confirm ease of comprehension and usability.

We previously described development of the BAST and how we established its content validity in English (Juengst, Terhorst, Dicianno, et al., 2019; Juengst, Terhorst, & Wagner, 2018; Osborne, Kauvar, & Juengst, 2019). In this article, we detail the iterative language validation process for the BAST and BAST Spanish version (BAST-ESP) in two phases. In doing so, we highlight the importance of stakeholder engagement and language validation to develop a self-reported measurement tool through rigorous scientific processes and to meet ethical guidelines of patient-centered research.

## Methods

### Phase 1: Adaptation for Lower-Literacy English

BAST content was derived from multiple previously validated outcome assessments measuring depression, anxiety, affect, behavioral dysregulation, aggression, coping, substance abuse, and fatigue (Juengst, Terhorst, Dicianno, et al., 2019). The items selected for inclusion in the BAST scored at an aggregated 12th grade literacy level using the Flesch-Kincaid grade level test (Flesch, 1948). Thus, the BAST developer (S.B.J.) rewrote each item to simplify the structure (e.g., removed double-barreled questions) and vocabulary (e.g., removing clinical jargon or high-level words) to make items more appropriate for people with cognitive deficits and/or low literacy. Rewritten items were further modified based on expert panel feedback (*n* = 7), focus group feedback from people with TBI (*n* = 11) and their family members (*n* = 10), and pilot testing with people with TBI (*n* = 162). The revised BAST achieved a Flesch reading score of 77.4 (equivalent to 8th grade), the level targeted by developers to be appropriate for adults with low-literacy (Flesch, 1948; Wang, Miller, Schmitt, & Wen, 2013)

### Phase 2: Translation, Language Validation, and Cognitive Interviewing for the BAST-ESP Adaptation

The content-validated, low-literacy English version of the BAST was the basis for the Spanish adaptation, termed the BAST-ESP (Juengst, Terhorst, Dicianno, et al., 2019). BAST survey items and instructions were translated into Spanish by a bilingual native Spanish speaker, and then back-translated to English by a bilingual native English speaker. The original and back-translated versions matched 78% of the time.

Investigators submitted the original English and Spanish translation to the University of Texas Southwestern Medical Center's Spanish Language Validation Resource (i.e., “the Committee”), which includes both native and academically qualified bilingual staff trained in language validation. The Committee assessed the readability scores of the BAST and BAST-ESP using the Simple Measure of Gobbledygook (SMOG) test, which performs most consistently among common readability metrics in health care contexts (Gilliam, Peña, & Mountain, 1980; McLaughlin, 1969; Wang et al., 2013). SMOG scores confirmed the low-literacy status of the BAST (9th-grade reading level) and the BAST-ESP (7th-grade reading level).

The Committee then reviewed the BAST and BAST-ESP to identify items for potential modification due to semantic, idiomatic, or conceptual concerns. Committee members, including the Spanish-speaking investigator (R.T.H.), met with the BAST developer (S.B.J.) to discuss concerns and recommend modifications to improve clarity and conceptual equivalence of both versions. A reassessment of literacy level after acceptance of the Committee's recommendations resulted in slightly lower grade level scores (8.8 English, 6.9 Spanish), improved readability, and greater semantic and conceptual equivalence.

Finally, the Committee validated completed language validation of the BAST-ESP using cognitive interviews. Cognitive interviewing is a more rigorous qualitative research technique for assessing participants' comprehension than traditional pilot testing alone (Berrigan et al., 2010; Carbone, Campbell, & Honess-Morreale, 2002; Lapka, Jupka, Wray, & Jacobsen, 2008; Willis, 2005) and is especially critical in adapting instruments from English to other languages (Berrigan et al., 2010; Lapka et al., 2008). Interviews probed specific words, concepts, and semantic structures, pinpointed areas of confusion, and solicited suggestions for adding to and improving survey language and thematic content. We recruited four Spanish-speaking people (who were originally from Mexico) with less than a high school education to participate in Spanish cognitive interviews. All participants provided informed consent to participate in cognitive interviews, in accordance with the protocol approved by the Institutional Review Board of the University of Texas Southwestern (STU 072017-083 Juengst).

## Results

**Table 1** traces specific examples of and rationales for the language modification, translation, and validation process of the BAST and BAST-ESP, including (1) improvements in clarity, (2) rephrasing of English idiomatic expressions, (3) reduction in literacy level, and (4) conceptual equivalence.

### English Literacy Adaptation Example

One original survey item intended to measure impulsive behavior asked participants to rate how often the following was true of them: “Thinking things through before acting (for example, consider finances before spending money).” This item was problematic for multiple reasons. First, it had a Flesch-Kincaid Grade level reading score of 11.7 (i.e., a 12th-grade reading level). Second, although the example of considering finances before spending money may seem potentially helpful for clarity, it could also lead a person to respond only to that specific example, particularly for people with cognitive impairments like perseveration and poor cognitive shifting. The item was initially rewritten in conjunction with the expert panel as, “I acted without thinking,” which earned a Flesch-Kincaid Grade level test score of 3.6. Members of the target population (i.e., people with TBI) recommended modifying the item to “I reacted without thinking,” as they believed this more accurately captured the intent of the item (i.e., impulsively reacting to something in the environment). Both items were then included in initial pilot testing, and factor analysis revealed that “I reacted without thinking” performed better (i.e., higher factor loading) than “I acted without thinking,” although the two were highly correlated. Therefore, we decided to keep “I reacted without thinking” in the BAST.

### Spanish Language Validation and Cognitive Interviewing Example

For the original English item, “I used drugs for non-medical reasons,” the word “drugs,” although pejoratively used for illicit drugs, was interpreted to include pharmaceutical medications by cognitive interview participants. However, the word “Drogas,” in Spanish, primarily referred to illicit drugs like cocaine or heroin, not capturing off-label use of prescription medication as intended by the original statement. To better capture abuse of medications like oxycodone or codeine, Spanish cognitive interview participants suggested a separate sentence, “Usaba medicamentos solamente por gusto o para razones no médicas” (“I used medications just for pleasure or for nonmedical reasons”). This distinction between recreational drugs and abuse of prescription medication was then applied to the original English, improving clarity and equivalence.

We also asked cognitive interview participants, “What stressors, if any, may uniquely apply to Spanish-speaking populations?” All four participants felt strongly that Latino people faced certain stressors not represented in the BAST for English speakers, including immigration status, family separation, and social discrimination. In addition, “too much work” (i.e., long work hours) was cited as a more relatable source of stress than “loss of employment.”

## Discussion

This article describes two important processes in the development of the BAST and BAST-ESP: (1) developing or adapting scientifically validated survey content for lower literacy and/or cognitively impaired English-speaking populations; and (2) using a systematic language validation process and cognitive interviewing to enhance the cultural appropriateness and the semantic, idiomatic, and conceptual equivalence for Spanish-speaking populations beyond Spanish translation alone. By enlisting Spanish speakers to participate in validation of the BAST-ESP, investigators confirmed that modifications achieved their intended outcome and were more inclusive of cultural considerations. The process also provided participants with TBI an opportunity to engage in research and contribute their feelings about the appropriateness and completeness of survey content. This inclusiveness of target populations exemplifies patient-centered research and strengthens the validity of our findings.

With no specific reference to brain injury, the BAST/BAST-ESP contributes a valuable tool that may be applicable to numerous clinical populations. Although the small number of participants in cognitive interviews limits our study, the consistency of responses among participants suggests that we may have achieved saturation in findings, and we intend further pilot testing and psychometric validation of the BAST-ESP in the future. This article, along with the BAST development article (Juengst, Terhorst, Dicianno, et al., 2019), may be considered a road map for other investigators to conduct systematic development or adaptation of scientific instruments for low-literacy and non–English-speaking populations. We recommend including stakeholders early and often through the assessment development process, pretesting survey materials with a sample similar to the target population, and employing the following strategies: phrase items in the first person (avoid passive verbs), phrase the entire question in each item, use single concepts for each item, use common words (avoid jargon), and strive for the fewest words possible.

## Figures and Tables

**Table 1:** Original Survey Adaptation to Develop BAST, Translation of BAST to BAST-ESP, Modifications, and Final Validation of BAST and BAST-ESP

**Original Items from Various Surveys** **Literacy Level: 12th Grade**	**Phase 1**		**Phase 2**		**Rationale for Modification**
	**BAST** **Literacy Level: 9th Grade**	**BAST-ESP Translation** **Literacy Level: 7th Grade**	**BAST-Validated** **Literacy Level: 8.8th Grade**	**BAST-ESP-Validated** **Literacy Level: 6.9th Grade**	
Sometimes I fly off the handle for no good reason	I got mad easily	Me enojaba fácilmente	No change	No change	Eliminated idiomatic expressed and simplified syntax
When I encounter a difficult, stressful, or upsetting situation, I “freeze” and do not know what to do	Do you feel stressed? [branching logic] When stressed, I was unable to make decisions	¿Se siente estresado? Cuando estaba estresado, no podía *hacer* decisiones	No change	¿Se siente estresado? Cuando estaba estresado, no podía tomar decisiones.	Simplified syntax and only asked when individuals endorsed stress, per focus group results “Tomar” is the best word for making decisions
Please check the response that corresponds to your answer	Then, *circle* the response that corresponds to your answer	Luego, *circula* la respuesta que corresponda a la pregunta	Then, *choose* your answer	Luego, escoja su respuesta	“Check” was odd without a checkbox “Circle” is appropriate for a paper survey, but “choose” is better for a mobile health app
Never Rarely Sometimes Frequently Always	Never Rarely Sometimes *Frequently* *Always*	Nunca *Raro* A veces *Frecuentemente* *Siempre*	Never Rarely Sometimes *Often* *Very often*	Nunca *Rara vez* A veces *Seguido* *Muy seguido*	The modified scales were previously validated and used as best practice for frequency Likert scales
Derived from multiple items^a^	When something upset me, I *had a hard time letting it go*	Cuando algo me molestaba, *era difícil olvidarme de ello*	When something upset me, *I kept thinking about it*	Cuando algo me molestaba, *seguía pensando en eso*	Translation of “letting it go” as “olvidarme” (forget) was deemed conceptually nonequivalent. “Kept thinking about it” translates equally and preserves the original survey items' intention
Derived from multiple itemsa	I used *strategies* in my day to day life	Usaba *estrategias* en *mi días cotidianos*	I used *coping* strategies in my day-to-day life	Usaba *estrategias para superar mi* vida día a día	Adding “coping” to “strategies” increases clarity
Apologize for misbehavior (for example, apologize for swearing)	I apologized when I did something wrong	*Pedía perdón* cuando hacía algo malo	No change	*Me disculpaba* cuando hacía algo malo	“Me disculpaba” (apologized) is a closer translation than “pedía perdón” (asked for forgiveness)
If somebody hits me, I hit back	I was *able* to walk away from a fight	Podía *alejarme* de una pelea	I *could walk* away from a fight	Podía *salirme* de una pelea	English: “could” is simpler than “able to.” Spanish: “salirme” (remove myself) is closer to “walk away from” than “alejarme” (distance myself)
Investigator created item^a^	I used drugs for non-medical reasons	Usaba *drogas sin* razón médica	Two items: *I used recreational drugs. I used medications not according to prescription*	Two ítems: *Usaba drogas. Usaba medicamentos solamente por gusto o para razones no médicas*	Split to 2 sentences given conceptual differences. “Drogas” connotes only illicit drugs in Spanish (e.g., cocaine), whereas “drugs” can sometimes mean prescription medicines in English so we added “recreational” in English. Language for prescription medication abuse suggested by cognitive interview participants in English and Spanish
Laugh or cry easily	I was *quick* to laugh or cry	Lloraba o reía *muy rápido*	I laughed or cried *easily*	Me reía o lloraba *fácilmente*	“Easily” and “fácilmente” are closer to the intended English colloquialism “quick to”
Recommended by consumer focus group^a^	Thoughts *got stuck in my head, and I could not stop thinking about them*	Pensamientos *se quedaban en mi cabeza y no podía parar de pensar en ellos*	No change	Los pensamientos se me quedaban en *mi mente* y no podía *dejar* de pensar en ellos	“Got stuck” didn't translate well; “se me quedaba” (stayed in) translated well. “Mi mente” (my mind) was preferable to “my head” for Spanish participants, and “dejar” (also ‘stop’) was preferable in cognitive interviews
Example: “The following are ways people react to various difficult, stressful, or upsetting situations”	Instructions: Please answer the following questions *to give us a better understanding of the current stresses or problems you have experienced*	Instrucciones: Por favor contesta la siguiente encuesta *para darnos un mejor entendimiento de su situación de estrés o problemas que haiga experimentado*	Instructions: Please answer the following questions *so we can better understand your recent experiences*	Instrucciones: Por favor conteste las siguientes preguntas *para que podamos entender mejor sus experiencias recientes*	The revision reduces the wordiness and thus the literacy level of the original
Investigator created item^a^	Marriage or *marital reconciliation*	Casamiento, o reconciliación *conyugal*	Marriage or *getting back together*	Casamiento, o *juntarse otra vez*	English: “Getting back together is simpler than “marital reconciliation.” Spanish: “en el matrimonio” is simpler than “conyugal.” For “getting back together”, “juntarse otra vez” is better than “reconciliación,” which is too formal and high lit
Investigator created item^a^	Are you being treated by a doctor, counselor, rehabilitation therapist, or other professional or *do you feel you need additional support?*	¿Estas siendo tratado por un doctor, consejero, terapeuta de rehabilitación, o algún otro profesional *o sientes que necesitas apoyo adicional?*	Are you being treated by a doctor, counselor, rehabilitation therapist, or other professional? *Do you feel you need additional support?*	¿Está recibiendo tratamiento por un doctor, consejero, terapeuta de rehabilitación, u otro profesional? *¿Usted siente que necesita apoyo adicional?*	Splitting the long sentence into two shorter sentences reduced the literacy level. Spanish cognitive interviews confirmed that the two shorter sentences were understandable
Modified and added items related to sources of stress	*Loss of employment* *Promotion in employment* *Increase in financial stress* *Major personal injury/illness*	-	*Not enough work* *Too much work* *Financial stress* *Personal injury/illness*	*No suficiente trabajo* *Demasiado trabajo* *Estrés económico* *Lesión o enfermedad personal* Added: *Racismo u otras formas de discriminación social* *Preocupación o temor relacionado al estado migratorio/ciudadanía* *Separación familiar*	Participants in cognitive interviews felt that promotion in employment was not stressful; rather, long work hours or not having enough work (and therefore enough money) was stressful. Thus proposed change from “loss/promotion” to “too little/too much work.” Changed “increase in” in financial stress to just “financial stress” because the stress was reportedly chronic. Similarly, the personal injury/illness need not be “major”; participants reported stress from chronic health problems. Lastly, participants in Spanish cognitive interviews reported sources of stress they felt were uniquely applicable to Spanish-speaking populations; these were added to BAST-ESP

Note. Items in italics represent changes across phases. BAST = Behavioral Assessment Screening Tool; ESP = Spanish.

a This is a comment/note added by the authors. It is not an original item from another survey.
